# Concordance Between E/e’ Ratio and N-Terminal Pro-B-type Natriuretic Peptide Levels for Predicting Post-Discharge Heart Failure Events Within Ninety Days After Acute Coronary Syndrome

**DOI:** 10.14740/cr2239

**Published:** 2026-08-31

**Authors:** Pannathorn Tangkongpanich

**Affiliations:** Division of Cardiology, Department of Medicine, Phramongkutklao Hospital, Bangkok, Thailand. Email: pannathorn.tan@pcm.ac.th

**Keywords:** Acute coronary syndrome, Heart failure, E/e’ ratio, NT-proBNP, Prognosis, Retrospective cohort study

## Abstract

**Background:**

Heart failure (HF) is a common complication after acute coronary syndrome (ACS) and is linked to substantial morbidity and mortality. Both the E/e’ ratio and N-terminal pro-B-type natriuretic peptide (NT-proBNP) are widely utilized as noninvasive signs of cardiac filling pressure and hemodynamic stress. This study aimed to determine whether concordance between the E/e’ ratio and NT-proBNP level was associated with post-discharge HF events within 90 days among patients hospitalized with ACS.

**Methods:**

The current retrospective cohort study included 195 adults with ACS admitted to a military and teaching hospital for the Royal Thai Army between July 1, 2021, and August 31, 2025. Patients were sorted into three groups according to the concordance of the E/e’ ratio and age-specific NT-proBNP thresholds: concordantly low, discordant, and concordantly high. The primary outcome was a post-discharge HF event within 90 days after discharge, defined as an outpatient or emergency visit requiring diuretic therapy or hospitalization for HF. Time-to-event analyses were done using Kaplan–Meier methods and Cox proportional hazards regression.

**Results:**

Of the 195 patients, 34 (17.4%) were in the concordantly low group, 69 (35.4%) in the discordant group, and 92 (47.2%) in the concordantly high group. Post-discharge HF events within 90 days occurred in 33 patients (16.9%). Event rates were 0.68, 1.64, and 3.21 per 1,000 person-days in the concordantly low, discordant, and concordantly high groups, respectively. Compared with the concordantly low group, the concordantly high group had a significantly higher risk of the primary outcome in univariable analysis (hazard ratio (HR): 4.50; 95% confidence interval (CI), 1.06–19.14; P = 0.042) and in the reported multivariable model (adjusted HR: 5.97; 95% CI, 1.22–29.10; P = 0.027). The discordant group did not have a statistically significant increase in risk.

**Conclusions:**

Concordant elevation of the E/e’ ratio and NT-proBNP identified patients with ACS at substantially higher risk of post-discharge HF events within 90 days. Concordant elevation of E/e’ and NT-proBNP was associated with a higher rate of post-discharge HF events within 90 days after ACS. These exploratory findings require validation in larger prospective multicenter studies.

## Introduction

Acute coronary syndrome (ACS) remains a leading cause of cardiovascular morbidity and mortality worldwide [1, 2]. Even after successful treatment of the index ischemic event, a substantial proportion of patients develop early decompensated or recurrent heart failure (HF), which contributes to rehospitalization and short-term adverse outcomes [1, 3–6]. Early identification of patients at increased risk of post-discharge HF may therefore support closer follow-up and timely optimization of guideline-directed therapy.

The E/e’ ratio derived from Doppler echocardiography is a practical surrogate marker of left ventricular filling pressure and diastolic burden [7–9]. N-terminal pro-B-type natriuretic peptide (NT-proBNP) is a well-established biomarker of myocardial wall stress and has diagnostic and prognostic value in HF and ACS [3, 4, 10–12]. Although each marker is clinically useful on its own, evidence is more limited regarding whether their concordant abnormality identifies a subgroup with particularly high vulnerability to subsequent HF events after ACS.

This study aimed to determine whether concordance between the E/e’ ratio and NT-proBNP level was associated with post-discharge HF events within 90 days among patients hospitalized with ACS.

## Materials and Methods

### Study design and setting

This study was conducted at the military and teaching hospital of the Royal Thai Army as a retrospective cohort study. The patients were identified from the electronic medical record database and the digital echocardiography archive. The study was approved by the Institutional Review Board of the Royal Thai Army Medical Department (Approval No. IRBRTA 1619/2568). The requirement for informed consent was waived because anonymized retrospective data were used.

### Study population

Eligible patients were adults aged 20 years or older who were diagnosed with ACS (ST-segment elevation myocardial infarction, non–ST-segment elevation myocardial infarction, or unstable angina), and had both NT-proBNP measurement and transthoracic echocardiography with E/e’ assessment conducted within 72 h of admission.

Patients were excluded if they had stage V chronic kidney disease (estimated glomerular filtration rate (eGFR) < 15 mL/min/1.73 m^2^), end-stage renal disease requiring regular hemodialysis, pregnancy, a mechanical prosthetic mitral valve, or severe mitral annular calcification that could compromise E/e’ assessment.

NT-proBNP measurement and transthoracic echocardiography were performed as part of routine clinical care within 72 h of admission. The timing was not protocolized. Exact timing relative to symptom onset, coronary angiography or percutaneous coronary intervention (PCI), administration of diuretics, and hemodynamic stabilization was not consistently retrievable from the retrospective records; consequently, between-group comparability of measurement timing could not be formally evaluated.

### Echocardiographic assessment

E/e’ values were obtained from finalized clinical echocardiography reports. The extracted dataset did not consistently retain separate septal and lateral e’ measurements; therefore, the precise annular sampling method could not be verified for every patient.

Patients with a mechanical prosthetic mitral valve or severe mitral annular calcification were excluded. Atrial fibrillation, significant mitral regurgitation, and regional wall-motion abnormalities were not systematically adjudicated as separate E/e’ reliability strata in the original dataset.

### Definitions and study groups

Elevated E/e’ was defined as E/e’ ≥ 14. NT-proBNP was categorized as elevated using the following age-specific thresholds: ≥ 450 pg/mL for patients aged < 50 years, ≥ 900 pg/mL for patients aged 50–75 years, and ≥ 1,800 pg/mL for patients aged > 75 years. These thresholds were based on the age-stratified diagnostic rule-in cutoffs validated in the International Collaborative of NT-proBNP Study by Januzzi et al and were originally developed to support the diagnosis of acute HF in patients presenting with acute dyspnea [13]. In the present study, these thresholds were used solely to define the NT-proBNP component of the concordance groups and were not applied as stand-alone diagnostic criteria for HF or as previously validated prognostic cutoffs after ACS.

### Outcomes

The primary outcome was defined as a post-discharged HF event within 90 days after discharge, defined as an outpatient department visit or emergency room visit requiring diuretic therapy, or hospitalization due to HF. On the other hand, secondary outcomes included all-cause mortality within 90 days and hospitalization for HF.

Post-discharge HF events were ascertained from outpatient, emergency department, and inpatient medical records. An event required documentation of HF by the treating physician together with initiation or intensification of diuretic therapy, or hospital admission primarily attributed to HF. Events were not adjudicated by an independent blinded committee, and reviewers were not formally blinded to the index clinical data.

### Statistical analysis

Categorical variables were summarized as frequencies and percentages and were compared by Chi-square test or Fisher’s exact test as appropriate. Continuous variables were expressed as mean ± standard deviation or median with interquartile range and were compared by one-way analysis of variance or Kruskal–Wallis test, depending on distribution [14].

Time-to-event analyses were conducted based on Kaplan–Meier methods with log-rank testing. Cox proportional hazards regression was used to estimate hazard ratios (HRs) and 95% confidence intervals (CIs) for post-discharged HF events. A two-sided P value < 0.05 was deemed statistically significant.

The number of covariates was constrained by the small number of primary events. Additional clinically relevant variables, including Killip class, prior HF, atrial fibrillation, ACS type, revascularization status, and pulmonary congestion, were not simultaneously entered because doing so would further increase model instability and overfitting. Some of these variables were also incompletely captured in the retrospective dataset.

## Results

### Baseline characteristics

An overall total of 195 participants met the eligibility criteria. Of these, 146 (74.9%) had non–ST-segment elevation myocardial infarction, 48 (24.6%) had ST-segment elevation myocardial infarction, and one patient (0.5%) had unstable angina. Thirty-four patients (17.4%) were classified as concordantly low, 69 (35.4%) as discordant, and 92 (47.2%) as concordantly high.

Patients in the concordantly high group were older, had lower renal function, more advanced Killip class, and lower left ventricular ejection fraction (LVEF) than patients in the other groups. Use of sodium-glucose cotransporter-2 inhibitors and mineralocorticoid receptor antagonists at discharge was also more frequent in the concordantly high group. Table 1 shows these data.

**Table 1:** Baseline Clinical Characteristics According to Concordance Between E/e’ Ratio and NT-proBNP Level

Characteristic	Concordantly low (n = 34)	Discordant (n = 69)	Concordantly high (n = 92)	P value
Age, years	61.5 (13.4)	68.6 (13.1)	71.7 (12.9)	< 0.001*^a^
BMI, kg/m^2^	25.6 (3.5)	24.5 (4.6)	24.7 (4.7)	0.458^a^
Hypertension	23 (67.6%)	54 (78.3%)	75 (81.5%)	0.248^b^
Dyslipidemia	23 (67.6%)	53 (76.8%)	72 (78.3%)	0.454^b^
Diabetes mellitus	13 (38.2%)	41 (59.4%)	46 (50.0%)	0.122^b^
Coronary artery disease	9 (26.5%)	15 (21.7%)	25 (27.2%)	0.719^b^
Chronic kidney disease	4 (11.8%)	23 (33.3%)	21 (22.8%)	0.050^b^
NSTEMI	23 (67.6%)	47 (68.1%)	76 (82.6%)	0.037*^c^
Killip class III–IV	2 (5.9%)	12 (17.4%)	23 (25.0%)	< 0.001*^b^
eGFR, mL/min/1.73 m^2^	79.9 (61.0, 92.1)	63.3 (40.0, 86.5)	60.4 (42.5, 81.1)	0.011*^d^
LVEF, %	50.0 (45.0, 60.0)	41.0 (30.0, 55.0)	34.0 (24.5, 42.0)	< 0.001*^d^
SGLT2 inhibitor at discharge	3 (8.8%)	23 (33.3%)	33 (35.9%)	0.011*^b^
Mineralocorticoid receptor antagonist at discharge	6 (17.6%)	20 (29.0%)	39 (42.4%)	0.021*^b^

Data are presented as n (%), mean (standard deviation), or median (interquartile range), as appropriate. P values with * are significant at alpha level of 0.05. ^a^One-way ANOVA; ^b^Chi-square; ^c^Fisher’s exact; ^d^Kruskal–Wallis. BMI: body mass index; NSTEMI: non–ST-segment elevation myocardial infarction; eGFR: estimated glomerular filtration rate; LVEF: left ventricular ejection fraction; SGLT2: sodium-glucose cotransporter 2; NT-proBNP: N-terminal pro-B-type natriuretic peptide; ANOVA: analysis of variance.

At discharge, sodium-glucose cotransporter-2 inhibitor use was 8.8%, 33.3%, and 35.9% in the concordantly low, discordant, and concordantly high groups, respectively. Mineralocorticoid receptor antagonist use was 17.6%, 29.0%, and 42.4%, respectively.

### Primary outcome

Post-discharge HF event occurred in 33 patients (16.9%) during the 90-day follow-up period. The event proportion increased across the three concordance groups, from 5.9% in the concordantly low group to 13.0% in the discordant group and 23.9% in the concordantly high group. Corresponding event rates were 0.68, 1.64, and 3.21 per 1,000 person-days, respectively. Primary outcome, in terms of the occurrence of late-onset HF, is presented in Table 2.

**Table 2:** Post-Discharge Heart Failure Events Within 90 Days

Outcome	Total	Concordantly low	Discordant	Concordantly high
Post-discharged HF events, n (%)	33/195 (16.9%)	2/34 (5.9%)	9/69 (13.0%)	22/92 (23.9%)
Total person-days for primary outcome	15,302	2,943	5,495	6,864
Event rate per 1,000 person-days	2.16 (1.53–3.03)	0.68 (0.17–2.72)	1.64 (0.85–3.15)	3.21 (2.11–4.87)

The log-rank P value was 0.036 for post-discharged HF events. HF: heart failure.

**Figure 1:**
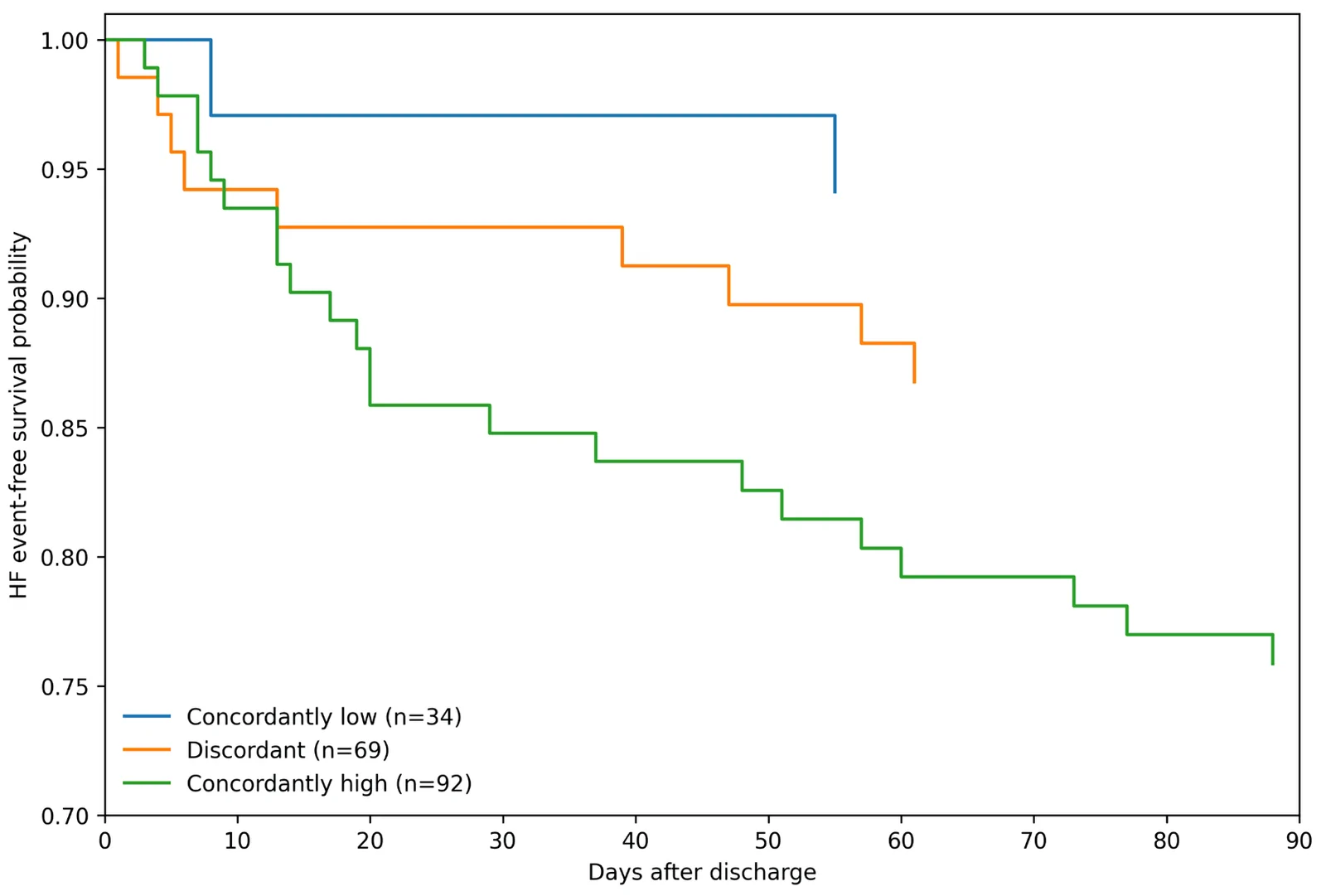
Kaplan-Meier curves for post-discharge heart failure events within 90 days stratified by concordance between the E/e’ ratio and NT-proBNP level. Numbers at risk at days 0, 30, 60, and 90 were 34, 33, 32, and 32 in the concordantly low group; 69, 62, 59, and 58 in the discordant group; and 92, 78, 72, and 68 in the concordantly high group, respectively. NT-proBNP: N-terminal pro-B-type natriuretic peptide; HF: heart failure.

The Kaplan–Meier analysis showed a significant difference in event-free survival among the groups (log-rank P = 0.036). As shown in Figure 1, patients in the concordantly low group had the most favorable event-free survival during follow-up, whereas those in the discordant group had an intermediate prognosis. Patients in the concordantly high group exhibited the poorest event-free survival, with a steeper and more sustained decline across the 90-day period, reflecting a greater risk of late-onset HF events after discharge.

Using univariable Cox regression, the concordantly high group had a significantly increased risk of post-discharged HF events compared with the concordantly low group (HR: 4.50; 95% CI, 1.06–19.14; P = 0.042). The discordant group did not show a statistically significant increase in risk (HR: 2.36; 95% CI, 0.51–10.93; P = 0.271). In the reported multivariable model, the concordantly high group remained significantly related to the primary outcome (adjusted HR: 5.97; 95% CI, 1.22–29.10; P = 0.027). Table 3 presents the Cox regression results for the primary outcomes.

**Table 3:** Cox regression Analysis for Post-Discharge HF Events Within 90 Days

Group	Univariable HR (95% CI)	P value	Reported adjusted HR (95% CI)	P value
Concordantly low	Reference		Reference	
Discordant	2.36 (0.51–10.93)	0.271	2.40 (0.49–11.73)	0.279
Concordantly high	4.50 (1.06–19.14)	0.042	5.97 (1.22–29.10)	0.027

The reported multivariable model was adjusted for age category, sex, eGFR, LVEF, sodium-glucose cotransporter-2 inhibitor use, and mineralocorticoid receptor antagonist use. HF: heart failure; HR: hazard ratio; CI: confidence interval; eGFR: estimated glomerular filtration rate; LVEF: left ventricular ejection fraction.

Given the limited number of primary outcome events relative to the number of model parameters, the multivariable model may have been overfit. Therefore, the adjusted hazard ratios should be interpreted as exploratory and may be unstable.

### Secondary outcomes

All-cause mortality occurred in six patients and HF hospitalization occurred in 17 patients. Although the crude frequencies were highest in the concordantly high group, the very small number of events resulted in substantial statistical uncertainty. These secondary analyses are descriptive and should not be used to draw definitive between-group conclusions.

HF hospitalization, a more objective component of the composite endpoint, occurred in 17 patients (8.7%): 2.9% in the concordantly low group, 7.3% in the discordant group, and 12.0% in the concordantly high group (Table 4). Because only 17 hospitalizations occurred, these findings are descriptive and should be interpreted cautiously.

**Table 4:** Secondary Outcomes

Outcome	Total	Concordantly low	Discordant	Concordantly high
All-cause mortality, n (%)	6/195 (3.1%)	0/34 (0.0%)	2/69 (2.9%)	4/92 (4.4%)
HF hospitalization, n (%)	17/195 (8.7%)	1/34 (2.9%)	5/69 (7.3%)	11/92 (12.0%)

Log-rank P values were 0.046 for all-cause mortality, and 0.028 for HF hospitalization. HF: heart failure.

## Discussion

In this retrospective cohort of patients hospitalized with ACS, concordant elevation of NT-proBNP and the E/e’ ratio identified a subgroup at markedly increased risk of post-discharged HF events after discharge. The risk gradient across concordantly low, discordant, and concordantly high groups supports the clinical relevance of integrating hemodynamic and biomarker-based indicators rather than relying on either measure alone.

The discordant group demonstrated an intermediate event rate and a point estimate above unity (HR = 2.36), although the association was not statistically significant and the CI was wide. This finding should not be interpreted as evidence of no risk. With a larger sample and more events, a clinically meaningful association in the discordant group might become statistically detectable. Discordance between E/e’ and NT-proBNP may reflect differences in measurement timing, age, renal function, transient hemodynamic conditions, treatment before measurement, or limitations of either marker. Therefore, an abnormal E/e’ with a low NT-proBNP, or the reverse pattern, should prompt integrated assessment of symptoms, physical findings, renal function, volume status, LVEF, and the overall echocardiographic profile rather than reliance on either marker alone.

These findings are biologically plausible. The E/e’ ratio correlates with left ventricular filling pressure and reflects diastolic burden, while NT-proBNP reflects myocardial stretch and wall stress [7–9]. Prior studies have shown prognostic value for natriuretic peptides and tissue Doppler parameters in patients with myocardial infarction or chronic HF [11, 12, 15 16]. The present study extends that literature by showing that concordant abnormality in both parameters identifies patients at particular risk for HF events soon after discharge.

The concordantly high group was older and had lower eGFR, lower LVEF, and a greater proportion of Killip class III–IV patients. Accordingly, the observed association may partly reflect greater baseline disease severity, renal dysfunction, and hemodynamic compromise rather than an independent effect of biomarker–echocardiographic concordance. Although selected variables were included in the reported adjusted model, residual confounding cannot be excluded.

This study has practical implications for post-ACS care. Because both NT-proBNP measurement and standard echocardiography are commonly available during the index hospitalization, their combined interpretation may help clinicians identify patients who need closer follow-up, tighter volume assessment, and earlier optimization of HF-directed therapies after discharge.

### Limitations

Several limitations should be acknowledged. First, this was a retrospective, single-center study, which is susceptible to selection bias, incomplete documentation, and residual confounding. Second, the total sample size was modest, and only 33 primary outcome events occurred; therefore, the precision of the effect estimates was limited, as reflected by the wide CIs. Third, the timing of biomarker and echocardiographic assessment was not standardized and may have influenced both NT-proBNP and E/e’ values during the acute phase of ACS. Finally, the present study evaluated the association between a concordance-based classification and post-discharge HF events but did not formally compare model discrimination, calibration, or fit against E/e’ alone, NT-proBNP alone, LVEF, Killip class, or a conventional clinical model. Therefore, incremental predictive value was not established. The findings should be regarded as exploratory and hypothesis-generating rather than confirmatory.

### Conclusions

In this retrospective cohort of patients with ACS, concordant elevation of the E/e’ ratio and NT-proBNP level identified a subgroup at markedly increased risk of post-discharged HF events within 90 days after hospital discharge. The observed gradient in risk across concordantly low, discordant, and concordantly high groups supports the clinical value of integrating these two noninvasive markers for early post-ACS risk assessment. Given their routine availability in contemporary practice, combined evaluation of E/e’ and NT-proBNP may guide intensity of follow-up and facilitate earlier optimization of evidence-based HF management. These findings identify an association that warrants prospective validation; they do not establish that the concordance strategy independently improves prognosis or should directly determine discharge planning or HF treatment. These findings should be considered exploratory and hypothesis-generating and require validation in larger prospective multicenter studies before clinical implementation.

## Data Availability

The deidentified analytic dataset may be available from the corresponding author upon reasonable request, subject to institutional approval and the limitations of archived retrospective records.
